# Supplementary material for: G4-binding drugs, chlorpromazine and prochlorperazine, repurposed against COVID-19 infection in hamsters
Source: Front Mol Biosci. 2023 Mar 16;10:1133123. doi: 10.3389/fmolb.2023.1133123 (PMC10061221; doi:10.3389/fmolb.2023.1133123)
Supplement: Supplementary file 1 [file DataSheet1.PDF]

Figure S1

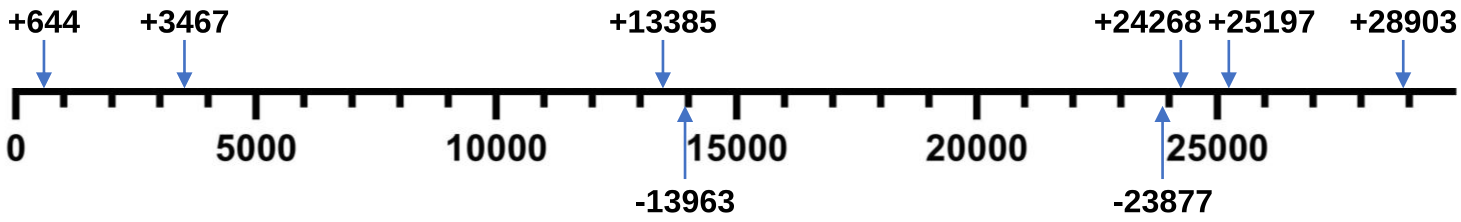

Figure S1: Representative scheme of the SARS-CoV-2 genome with the positions of some of the pG4s studied so far marked with arrows (Zhang et al., 2020; Bezzi et al., 2021; Ji et al., 2021; Zhao et al., 2021).

Table S1

PQS	Sequence	Genomic Position	cGcG	G4H	G4NN	pqsfinder
pG4-1	**GGG**UAUUAAAAUACAAGA**GGG**U**G**T**GG**UU**G**AUUAU**GG**	NC_045512v2:4,486-4,521	31	0.7778	0.0617	11
pG4-2	**GGG**UCA**GGG**UUUAAAU**GG**UUACACU**G**UA**G**A**GG**	NC_045512v2:4,255-4,286	10	0.7813	0.3162	19

Table S1: New pG4s identified in the SARS-CoV-2 genome that have bulges. Their sequences, genomic positions are mentioned along with the G4 propensity scores (cGcC- Consecutive G over consecutive C ratio, G4H- G4Hunter score, G4NN- G4 Neural Network, and pqsfinder score)

Figure S2

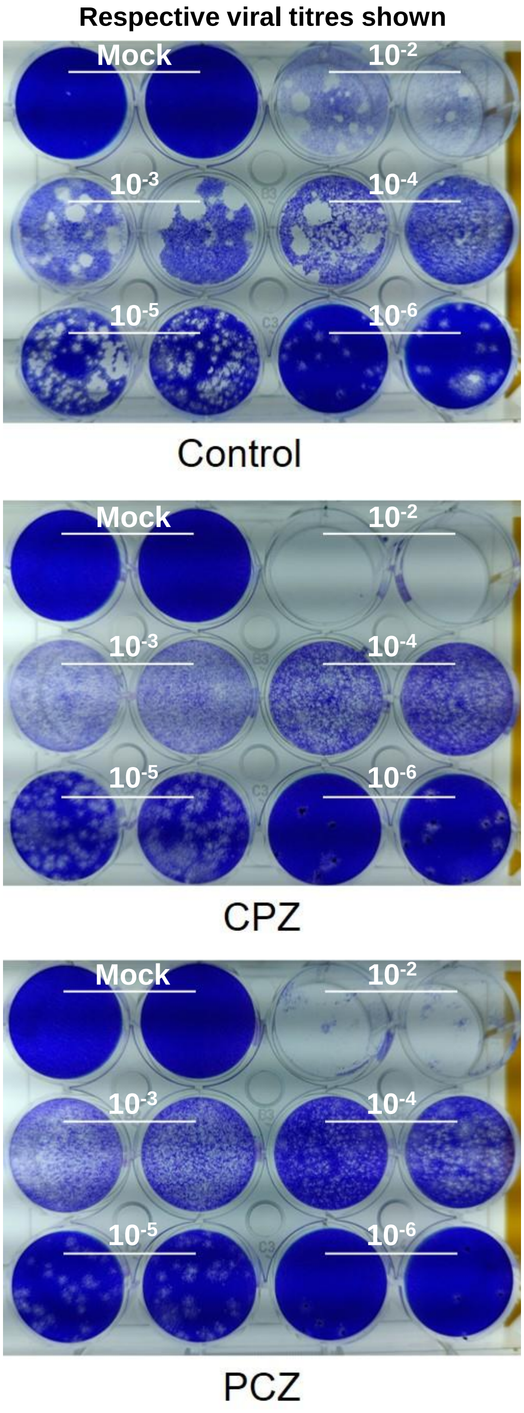

Figure S2: Representative PFU assay plates showing consistent drop in the plaques with logarithmic dilution of the sample and the effects on the number of PFUs after CPZ or PCZ treatment. Respective viral titres shown above the wells.

Figure S3

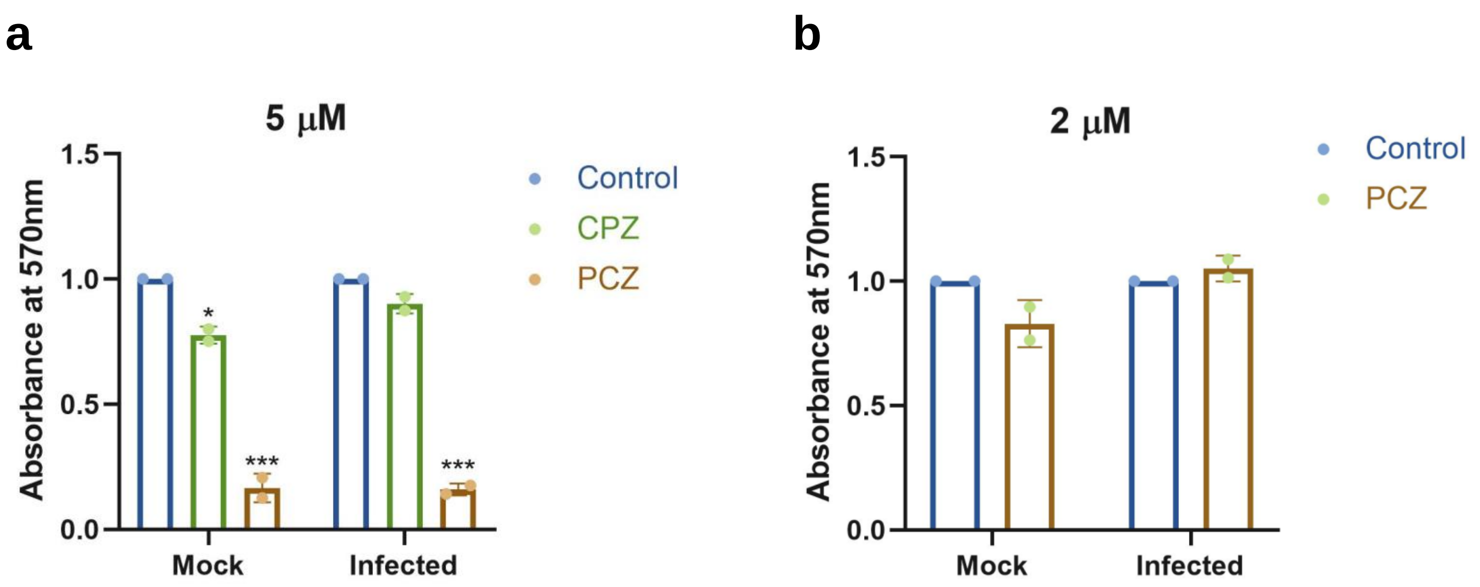

Figure S3: Viability of SARS-CoV-2 infected or uninfected Vero cells treated with 5 μM CPZ and PCZ (**a**) or 2 μM PCZ (**b**) compared against untreated control, measured by MTT assay. Mean ± SD (n=2); unpaired, two-tailed *t*-test.

Figure S4

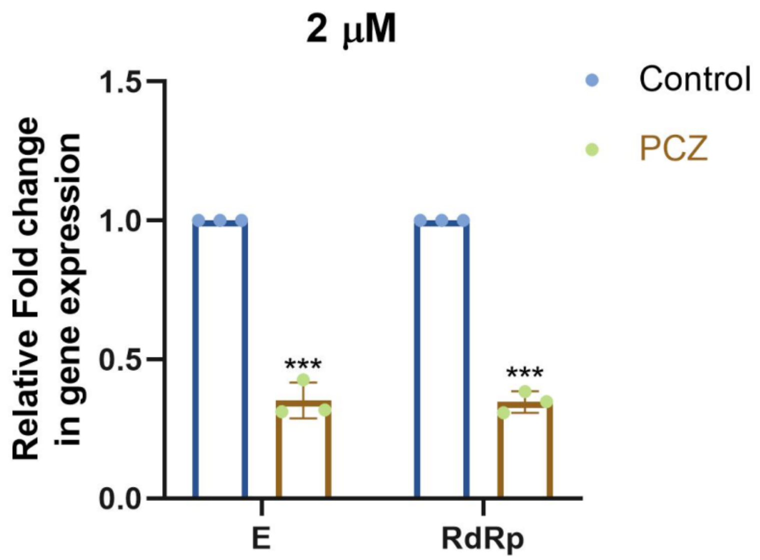

Figure S4: Expression of E and RdRp genes of SARS-CoV-2 in the extracellular media of infected Vero cells treated with 2 μM of PCZ compared against untreated control. Mean ± SD (n=3); unpaired, two-tailed *t*-test.
